# Accelerated Coronary Artery Disease in a Patient With Advanced Lung Cancer: Interplay of Cancer, Inflammation, and Treatment Effects

**DOI:** 10.7759/cureus.77769

**Published:** 2025-01-21

**Authors:** Oluwaremilekun Tolu-Akinnawo, Kayode E Ogunniyi

**Affiliations:** 1 Internal Medicine, Meharry Medical College, Nashville, USA; 2 Internal Medicine, University Hospital of North Durham, Durham, GBR

**Keywords:** cad, chemotherapy, immunotherapy, radiotherapy, small cell lung cancer

## Abstract

Cardiovascular disease (CVD) and lung cancer are among the most prevalent causes of death worldwide, representing substantial public health challenges. The relationship between coronary artery disease (CAD) and lung cancer is potentially multifaceted, influenced by common risk factors and the adverse cardiac effects of cancer treatments. However, cases of accelerated CAD occurring within a year of cancer treatment initiation are rarely reported. We present a case of a Black American male undergoing combination therapy (chemotherapy/radiotherapy/immunotherapy) for small-cell lung cancer, who developed ST-elevation myocardial infarction (STEMI) seven months after beginning treatment. Although we do not have the official records, the patient reported that he had previously undergone a cardiac workup (including an echo/stress test) at an outside facility a year prior, due to persistent dyspnea, which was unremarkable. The patient underwent successful percutaneous coronary intervention and was discharged on apixaban and prasugrel. This study underscores the importance of maintaining a high index of suspicion for acute coronary syndromes (ACS) in patients receiving lung cancer treatments, emphasizes the need for early recognition of warning signs, and highlights the critical role of risk factor management and enhanced surveillance in this vulnerable population.

## Introduction

Cardiovascular disease (CVD) and lung cancer are two leading causes of death globally, representing significant public health challenges [1]. CVD encompasses a wide range of conditions, including coronary artery disease (CAD), heart failure (HF), arrhythmias, valvular heart diseases, and chronic cardiovascular risk factors such as hypertension, diabetes, hyperlipidemia, and hypothyroidism. The global burden of CVD, in terms of both incidence and mortality, has been steadily increasing in recent decades, underscoring its critical public health impact [2]. Similarly, lung cancer is the second most common cancer worldwide and remains the leading cause of cancer-related deaths [3]. While CVD accounts for approximately 17.9 million deaths annually, lung cancer is responsible for 1.8 million deaths each year, emphasizing the immense toll of these conditions [4,5].

Despite advancements in early screening and technological innovation leading to a decline in lung cancer incidence and mortality, the intricate interconnection between CVD and lung cancer continues to be a major concern [6]. Both conditions share overlapping risk factors, such as smoking, aging, and inflammation, which contribute to their pathogenesis [7]. Moreover, treatments for lung cancer, including chemotherapy, immunotherapy, and radiation, are associated with cardiotoxicity, potentially exacerbating the risk of CVD [8].

Among the various forms of CVD, CAD is a particularly significant contributor to CVD-associated mortality. Observational studies have suggested a potential bidirectional association between CAD and lung cancer. However, there is limited evidence of the phenomenon of accelerated CAD occurring within seven months of diagnosis. Risk factors such as tobacco exposure can induce inflammatory mediators and reactive oxygen species, which promote DNA damage and the development of both cancer and CAD [9-11]. Tobacco also exacerbates CAD by reducing nitric oxide levels, leading to vasomotor dysfunction and increased oxidative stress [12]. In addition, cancer therapies - including chemotherapy, immunotherapy, and radiotherapy - have been linked to cardiotoxic effects [13]. Genetic factors, such as pleiotropic mutations in the DYRK1B gene, further highlight the shared molecular pathways underlying CVD and lung cancer and warrant closer examination in understanding their pathogenesis [14,15].

This study discusses a case of accelerated CAD (developing within seven months) in a patient with advanced lung cancer. It highlights the complex interplay of multiple risk factors, including radiotherapy, chemotherapy (etoposide), immunotherapy (durvalumab), and a history of tobacco use.

## Case presentation

A 62-year-old Black American male with a medical history of chronic obstructive pulmonary disease (COPD), hypertension, small-cell lung cancer, and a 30-pack-year smoking history (ceased 30 years ago) presented to the emergency department (ED) with acute chest pain. The chest pain had started 12 hours prior to the presentation and was described as mid-sternal, pressure-like, and squeezing in nature. The patient rated the pain as 8-9/10 in severity, with radiation to the left arm and jaw. The pain was transiently relieved by nitroglycerin and had no identifiable aggravating factors. Associated symptoms included shortness of breath (SOB) and occasional diaphoresis. The patient denied nausea, vomiting, palpitations, orthopnea, paroxysmal nocturnal dyspnea (PND), or leg swelling. Although we do not have the official records, the patient reported that he previously had a cardiac workup (including an echo/ stress test) done a year prior at an outside facility due to persistent dyspnea which was unremarkable. He also reported that he had been following up regularly for his annual physicals.

**Figure 1:**
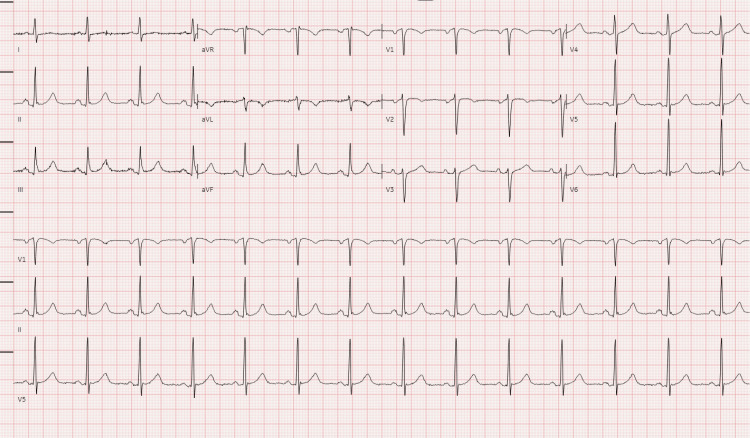
EKG at the initial presentation showing baseline changes. EKG: electrocardiography

Notably, the patient had visited the ED earlier that day for similar symptoms but was discharged after no acute findings were observed. Vital signs at that time were as follows: blood pressure (BP) of 124/76 mmHg, heart rate (HR) of 64 beats per minute, and oxygen saturation of 99-99% on room air. Initial investigations at that time included an electrocardiogram (EKG), which revealed normal sinus rhythm with a ventricular rate of 84 beats per minute (bpm), T-wave inversion in lead V2 (single lead - usually non-specific finding), and no ST-segment elevation or depression (Figure 1). Laboratory studies showed a troponin level of 62 ng/L, which increased to 63 ng/L six hours later (reference range: 0-20 ng/L), indicating a stable trend. Other notable findings included a glucose level of 108 mg/dL (reference range: 74-106 mg/dL), total cholesterol of 224 mg/dL (reference range: <200 mg/dL), and LDL cholesterol of 146 mg/dL (reference range: <100 mg/dL).

Due to the persistence of symptoms, he returned for further evaluation. On arrival, the patient’s vital signs were significant for a blood pressure of 167/110 mmHg, heart rate of 139 bpm (likely secondary to pain and ongoing ischemia), respiratory rate of 20 breaths/min, and oxygen saturation of 98% on room air. Physical examination revealed tachycardia but no murmurs, rubs, or gallops. The respiratory examination was unremarkable, with clear breath sounds bilateral. The abdominal and extremity examinations showed no abnormalities, including the absence of edema or signs of deep vein thrombosis (DVT).

**Figure 2:**
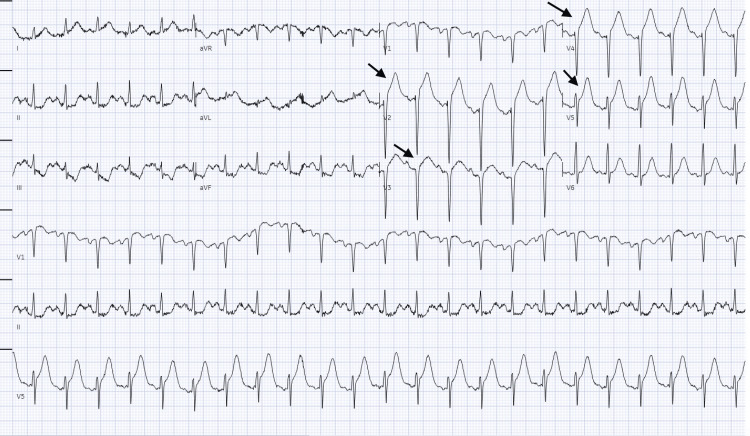
EKG showing ST elevation in the anterior leads (V1-V4) and reciprocal changes in the inferior leads (II, III, AVF). EKG: electrocardiography; AVF: arteriovenous fistula

Given the elevated troponin levels and persistent chest pain, a repeat EKG was obtained which revealed ST elevation in the anterior leads (V1-V4) and reciprocal changes in the inferior leads (II, III, arteriovenous fistula {AVF}) (Figure 2). This is a significant change compared to the initial visit.

**Figure 3:**
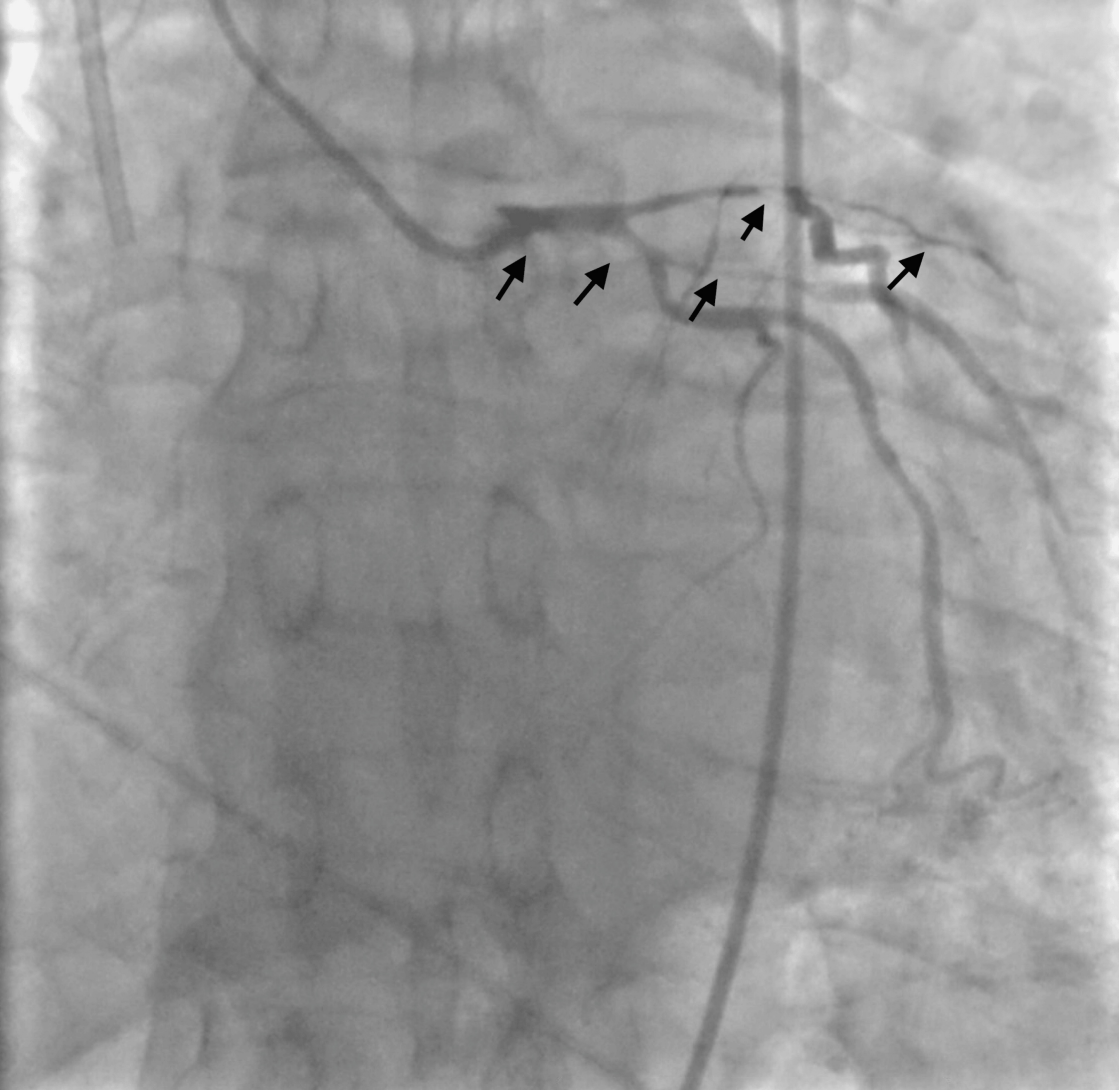
Catheterization with severe CAD (arrows). CAD: coronary artery disease

A cardiology consultation was obtained, an ST-elevation myocardial infarction (STEMI) protocol was activated, and the patient was transferred emergently to the catheterization laboratory. Coronary angiography revealed extensive thrombus in the proximal left anterior descending (LAD) artery, proximal circumflex artery, and high obtuse marginal (OM) branch (Figure 3).

**Figure 4:**
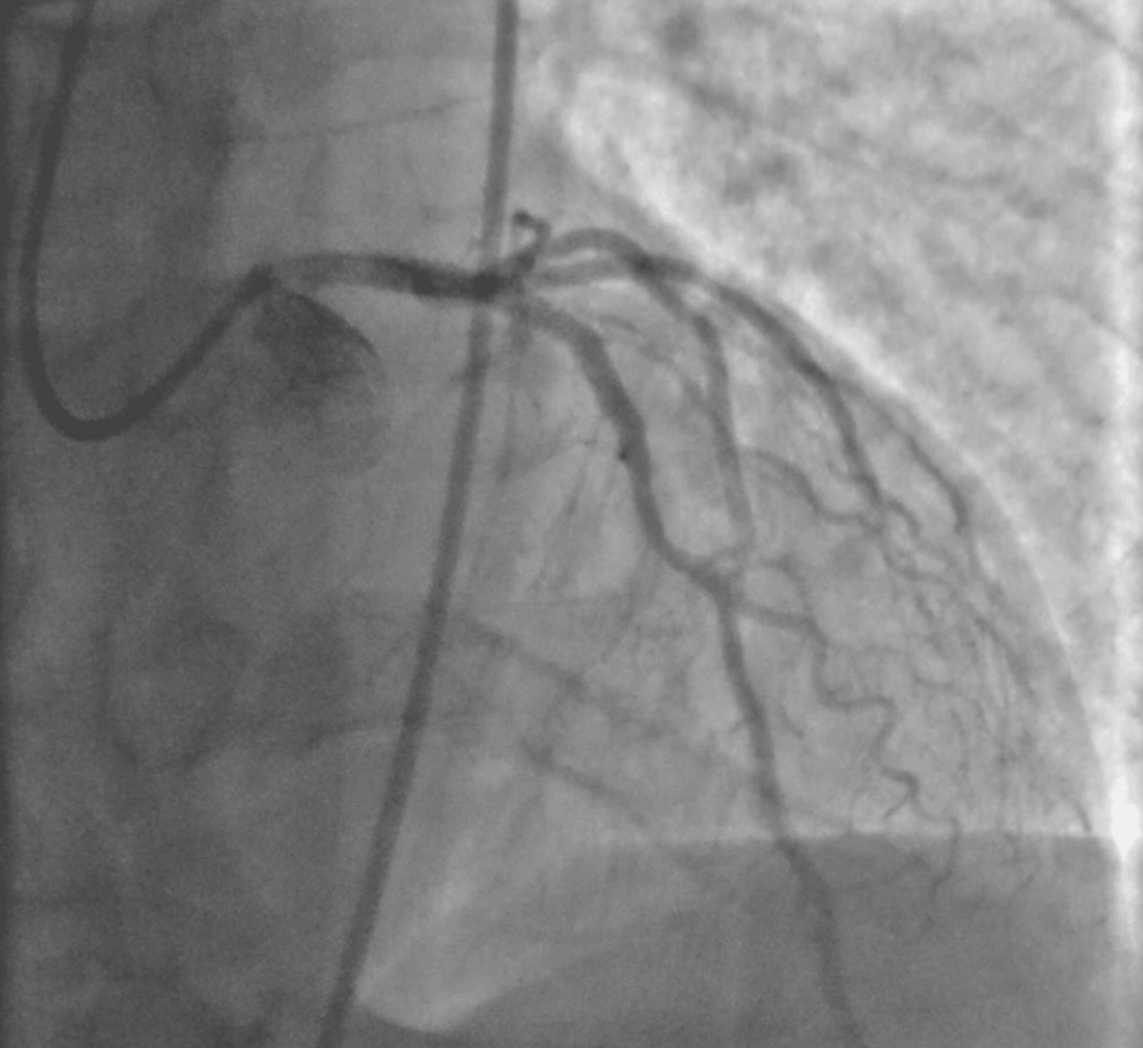
Post-intervention image with improved areas of occlusion.

**Figure 5:**
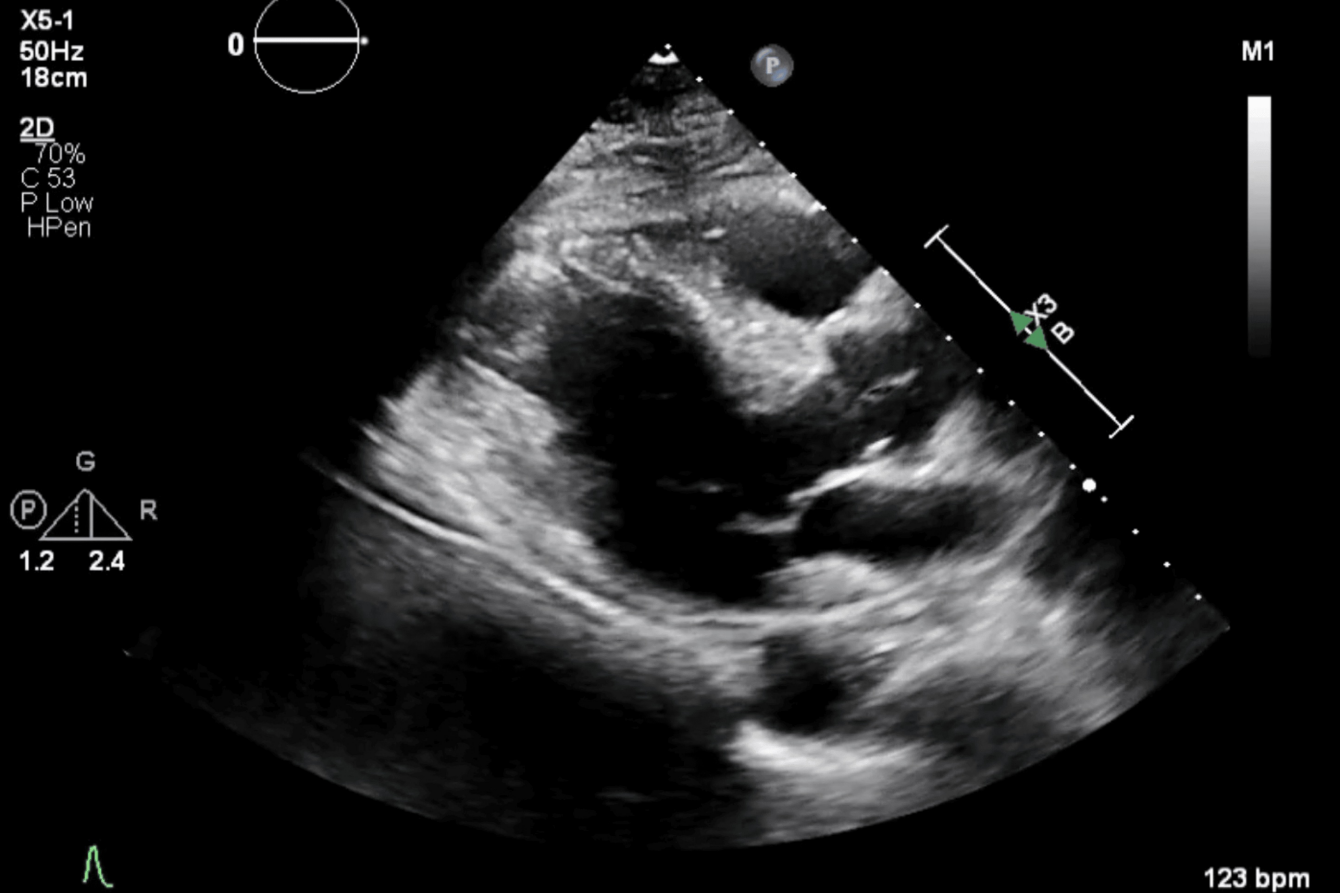
Post-procedural 2D echo with severely reduced EF. Echo: echocardiogram; EF: ejection fraction

Drug-eluting stents were successfully placed in the LAD and circumflex OM branches with excellent results (Figure 4). Post-procedure left ventricular ejection fraction (EF) was estimated at 25%, down from 30% to 35% on an echocardiogram (2D echo) performed two weeks earlier (Figure 5). Anterior wall hypokinesis was also observed.

The patient was stabilized in the intensive care unit (ICU) on a regimen of aspirin, prasugrel, and tirofiban. Goal-directed medical therapy was initiated to manage his blood pressure. A wearable defibrillator was placed for cardiac protection (due to the risk of sudden cardiac death usually from arrhythmias in patients with severely reduced ejection fraction {EF} as in the case of our patient), and he was discharged on apixaban and prasugrel due to a suspected hypercoagulable state secondary to malignancy and recent cancer treatments.

The patient’s oncological history is significant for a diagnosis of small-cell lung cancer in March 2023, following the identification of a left neck mass. Subsequent investigations revealed mediastinal lymphadenopathy and a biopsy confirmed small-cell lung cancer with neuroendocrine differentiation. Initial treatment included cisplatin, etoposide, and durvalumab, followed by radiation therapy, which was completed in June 2023. Maintenance therapy with durvalumab was initiated in July 2023. Imaging in August 2023 showed an excellent response to treatment with no evidence of active disease. However, by November 2023, follow-up imaging demonstrated stable mediastinal and hilar adenopathy with no new findings, alongside a few centrilobular nodules in the medial right lower lobe, likely inflammatory or infectious.

This case highlights the complex interplay of cardiovascular disease and malignancy, particularly in the context of hypercoagulability and cardiotoxicity induced by cancer therapies. The patient’s presentation of accelerated coronary artery disease (CAD) within seven months underscores the importance of vigilant monitoring in patients with concurrent oncological and cardiovascular comorbidities.

## Discussion

CAD and lung cancer

CAD and lung cancer continue to pose significant public health concerns due to the continued high rate of mortality. Interestingly, they also shared multiple risk factors, such as smoking, hypertension, type 2 diabetes mellitus (DM), advanced age, overweight, and socioeconomic status [16-19]. A population-based study found CAD to be one of the most prevalent CV conditions in patients with lung and bronchus cancer [20]. Also, a meta-analysis noted lung cancer as an independent risk factor for the development of CAD and myocardial infarction (MI) [21]. A low socioeconomic status (SES) (as in the case of our patient, a low-income person, limited education, currently unemployed) is also a well-documented risk factor for both lung cancer and CAD [16]. In the case of our patient, who developed accelerated CAD within seven months, notable risk factors include underlying lung cancer, lower SES, a smoking history, and the adverse cardiovascular effects of lung cancer therapy, including radiotherapy, each of which will be discussed extensively.

Pharmacological therapies for lung cancer and CAD

Studies have shown a potential correlation between antineoplastic regimens for lung cancer and the development of CAD. Immune checkpoint inhibitors, cytotoxic chemotherapy, and radiation have all been identified [21]. The present patient was commenced on cisplatin, etoposide, and durvalumab every three weeks for four cycles over three months (from 3/23) prior to developing CAD. He also underwent radiotherapy to the primary tumor for two months (April 2023 to June 23) and was subsequently started on durvalumab as maintenance therapy (7/23) four months prior to CAD. Each of these treatments has been identified in the literature as a potential risk factor for CAD. Below, we discuss how each of our patient’s risk factors could have contributed to the development of his accelerated CAD/MI. 

Chemotherapy (Etoposide/Cisplatin) and CAD

The development of chemotherapy agents has significantly advanced healthcare by reducing cancer-related morbidity and mortality; however, many cancer survivors face an increased risk of cardiac diseases, including coronary artery disease (CAD) [22]. Cancer treatment often involves a complex regimen of medications, radiation therapy, and surgical interventions, with chemotherapy and radiation identified as contributors to CAD risk [22,23].

Etoposide, a plant-alkaloid chemotherapy agent, works by disrupting DNA synthesis and inducing apoptosis in cancer cells. Etoposide inhibits DNA synthesis by forming a complex with topoisomerase II and DNA with the formed complex inducing breaks in the double-stranded DNA and preventing the repair by topoisomerase II binding. Accumulated breaks in DNA in turn prevent entry into the mitotic phase of cell division eventually leading to cell death. It is frequently used to treat refractory testicular tumors and small-cell lung cancer, as in the present case. While hypotension is the most common cardiac side effect, myocardial ischemia and infarction have also been reported [23]. Studies suggest a combined effect of etoposide and bleomycin in accelerating CAD [24]. For instance, a case of accelerated CAD occurred following single-agent etoposide use in a patient with persistent Hodgkin’s disease [25]. Similarly, a 32-year-old male receiving bleomycin, etoposide, and cisplatin for embryonal carcinoma developed myocardial infarction (MI) [26], and another patient undergoing cisplatin-etoposide treatment for bronchogenic cancer experienced suspected MI [27].

Cisplatin, a cytotoxic agent commonly used in lung cancer treatment, is strongly associated with thrombosis. Proposed mechanisms include direct endothelial damage, decreased anticoagulant protein C activity, elevated prothrombotic von Willebrand factor (vWF) levels, platelet activation via the arachidonic pathway, and hypomagnesemia, all of which increase thrombosis risk [28,29]. This elevated thrombosis risk has been linked to higher rates of acute coronary syndrome (ACS), particularly in patients over 65 years old and those receiving concurrent radiation therapy [21,30]. Cisplatin use is also associated with venous and arterial thromboembolism and late-onset left ventricular diastolic dysfunction [21]. Several studies highlight the cardiotoxic risks of cisplatin-based regimens. In a study of 21 testicular cancer patients treated with cisplatin, vinblastine, and bleomycin, 38% developed angina during therapy [31]. Similarly, in a cohort of 78 lung cancer patients treated with cisplatin and etoposide, three developed MI during chemotherapy [32]. Case reports describe young patients treated with bleomycin, etoposide, and cisplatin who subsequently developed acute MI [33]. Additionally, Lee et al. reported three cases of cardiotoxicity in patients undergoing combination chemotherapy with etoposide, cisplatin, and continuous 5-fluorouracil infusion, presenting with chest pain, tachycardia, and electrocardiographic changes indicative of MI or ischemia during treatment for advanced non-small cell lung cancer [34].

Durvalumab (Immunotherapy) and CAD

Although we believe the case of accelerated CAD in our patient is likely multifactorial, we however suspect durvalumab played a huge role in it as the patient was currently on a durvalumab maintenance cycle (as in the initial combined chemoimmunotherapy x 4 cycles, then maintenance for four months prior to CAD). Durvalumab belongs to a class of immunotherapy that acts via immune checkpoint inhibition. They are now gradually becoming the first-line treatment for patients with lung cancer with negative driver mutations [35]. Immune checkpoint inhibitors (ICIs) act by activating the immune system to eliminate cancer cells by targeting one of the following checkpoints: programmed death-1 (PD-1) as well as its ligand (PD-L1) as in the case of durvalumab, the lymphocyte activation gene 3 (LAG3), and the cytotoxic T-lymphocyte associated protein 4 (CTLA-4) [36]. Durvalumab is a PD-1 inhibitor and is often used in advanced or metastatic small-cell lung cancer as in the case of our patient. A study by Chitturi et al. analyzed major adverse cardiac events (MACEs) between patients receiving ICIs and non-ICIs. The study revealed that patients receiving ICIs are more likely to have cardiovascular (CV) death, fatal MI, and cardiac arrest [37]. In this study, MACEs among patients treated with ICIs (n=18) were predominantly related to cardiovascular death from myocardial infarction (n=13), cardiac arrest (n=6), and hospitalization for heart failure (n=4) in a patient with a non-fatal myocardial infarction. Other cardiovascular adverse effects included cardiac-related chest pain (n=25), arrhythmia and conduction abnormalities (n=25), cardiomyopathy, and heart failure (n=20) [37]. The median age for these events was 51 days which is shorter than our patient's exposure [37]. This was backed by another recent study that noted an three times increased risk of atherosclerotic CV events, including MI, and ischemic stroke in patients receiving ICIs for treatment [38]. Similarly, there is also an increased risk of aortic atherosclerotic changes and a concomitant increased risk of aortic stenosis in patients receiving ICIs [38]. Additionally, a recent systematic review with a meta-analysis of 48 randomized clinical trials also noted CV adverse events such as MI, and ischemic stroke among others were common among ICI use [39].

Radiotherapy and CAD

Several studies have shown an increased risk of CAD in patients with underlying lung cancer, more frequently studied are cases of non-small cell lung cancer (NSCLC). The proposed mechanism is direct damage to the endothelial lining of the coronary arteries leading to progressive inflammation, oxidative stress, and accelerated development of atherosclerosis. This process ultimately leads to the narrowing and thickening of coronary vessels (through the proliferation of smooth muscles and collagen deposition) and reduced cardiac blood supply due to the continuous generation of reactive oxygen species (ROS) from radiation exposure. This process also causes impaired repair mechanics within the damaged tissue further amplifying the process of atherosclerosis. Radiation-induced endothelial dysfunction and inflammation can also accelerate the development of atherosclerotic plaques, especially in patients with underlying CAD [40,41]. It is important to understand that the severity of radiation-induced CAD is dose-dependent and the development is delayed which could take months (as in the case of our patient) to years [40]. A study by Herbach et al. investigating the risk of cardiac events in patients receiving chemotherapy alone or chemoradiation for the treatment NSCLC noted that after 13 months of follow-up, 70 of 682 (10.26%) patients who received chemoradiation and 43 of 684 (6.30%) matched patients who received chemotherapy alone developed a severe cardiac event (p=0.008) [40]. Of these, 11 cases were sudden cardiac deaths, 23 developed acute MI, 16 were hospitalized with heart failure, <11 were admitted for ischemic heart disease other than acute MI, while 13 underwent percutaneous coronary intervention or coronary bypass graft procedure [40]. Also, chemoradiation increased the rate of severe cardiac events (cause-specific HR: 1.62; 95% CI: 1.11-2.37) as well as increased cancer severity (non-cardiac death cause-specific HR: 2.53; 95% CI: 1.93-3.33) [40].

Smoking and CAD

Although our patient was not currently smoking at the time of the incident MI, he still had a significant smoking history (~30 pack years) which we believe may have contributed to the development of CAD either directly or indirectly (through lung cancer). Smoking remains the single most important risk factor for the development of CAD. Smoking also remains the primary risk factor for premature coronary atherosclerosis by increasing the oxidation of low-density lipoprotein (LDL), while damaging coronary endothelial vasodilation. Both mechanisms lead to the development of CAD through premature and accelerated coronary atherosclerosis both of which are determinants of the severity and extent of vascular occlusion [42,43]. Smoking has many significant detrimental effects on the CV system such as reduction in nitric oxide leading to vasomotor dysfunction, increased prothrombogenic activities, increased oxidative LDL, increased inflammation as well as oxidative stress [44]. Not only that, but through arterial wall hardening, smoking also increases the risk of hypertension and insulin resistance, both of which have been identified as possible risk factors for the development of accelerated CAD [45,46]. The increased endothelial inflammation triggered by smoking is via the production of free radicals, reactive oxygen, and nitrogen species [47]. These processes leading to endothelial injury increase adhesion molecules and recruitment of immune cells, further reinforcing the inflammatory state [48]. Recurrent inflammation is also a percussion to the development of cancer which emphasizes a bi-directional pathway between CAD and lung cancer [49,50]. Due to these reasons, there is a need for higher suspicion of CAD, especially in patients with CAD and lung cancer with previous cigarette smoking as in the case of our patient.

CAD risk reduction in patients with lung cancer

Several studies have shown a possible association between chemoradiation agents for lung cancer and the incident development of CAD; however, few cases highlight acute MI with ongoing treatment just as in the case of our patient. This emphasizes the need for high suspicion as well as addressing modifiable risk factors to reduce the risk.

It is crucial that patients with underlying CV risk factors diagnosed with lung cancer need to be optimized prior to starting lung cancer treatments. One of the most critical interventions is smoking cessation. This is because smoking remains the most significant risk factor for the development of CVD and incident lung cancer. Smoking cessation could involve a combination of behavioral intervention as well as pharmacological [51]. In the case of our patient, he quit smoking 30 years prior to acute MI; however; a history of smoking still remains a risk factor. Also, underlying hypertension, diabetes mellitus, and hyperlipidemia, as in the case of our patient, need to be optimized, as poorly controlled levels increase the risk of accelerated CAD, which could have contributed in the case of our patient. The control of BP is important as poorly controlled hypertension has been shown to increase the risk of adverse cardiovascular events compared to other risk factors [20,51,52]. This should increase a triad management guideline of dietary modification, lifestyle modifications, and pharmacological intervention as needed.

Other risk factors that need to be addressed include thyroid disorders, sedentary lifestyle, and substance abuse, as well as the evaluation and treatment of hypercoagulable disorders. Patients with a family history of premature CAD should be screened for genetic predisposition, such as elevated lipoprotein(a) levels.

Screening and surveillance

The interplay between lung cancer and cardiovascular disease presents significant challenges in patient management, particularly given the well-documented association between cancer therapies and an increased risk of coronary artery disease (CAD) [53,54]. Close cardiovascular monitoring throughout cancer treatment is essential, and a proactive, multidisciplinary approach is critical to minimize adverse effects and complications. Coordinated care among oncology, cardiology, general medicine, radiology, pharmacy, and social work teams ensures comprehensive management that addresses both cancer progression and cardiovascular risks [55]. Oncologists tailor cancer treatment to balance therapeutic efficacy with potential cardiotoxicity. For example, if a specific chemotherapeutic agent is linked to myocardial infarction (MI), the oncologist may switch to a less cardiotoxic alternative. General practitioners oversee the overall care plan, ensuring age-appropriate screenings and appropriate management of coexisting conditions. Cardiologists provide targeted preventive and therapeutic strategies to manage CAD, while radiologists adjust imaging protocols and radiation dosing to minimize cardiovascular risks associated with thoracic irradiation. Pharmacists guide the choice and dosing of medications to avoid interactions between cancer therapies and cardiovascular treatments, optimizing the patient’s overall regimen. Social workers offer emotional support and practical resources, helping patients and families navigate healthcare systems, access financial aid, and manage the dual burden of cancer and cardiovascular disease [53-55].

Baseline cardiovascular risk assessments are crucial before starting lung cancer treatment. These assessments should include vital signs to evaluate blood pressure control, a complete metabolic panel to assess renal function and glucose levels, a lipid panel for hyperlipidemia, and hemoglobin A1C for diabetes screening. Monitoring should be individualized, with periodic evaluations tailored to underlying comorbidities. For example, patients with poorly controlled diabetes should have hemoglobin A1C measured at least every three months [54].

Certain cancer treatments require enhanced cardiovascular monitoring. Immune checkpoint inhibitors (ICIs), such as pembrolizumab, are associated with increased risks of myocarditis and myocardial infarction. Current recommendations advise baseline evaluations, including troponin levels, electrocardiograms (ECGs), and brain natriuretic peptide (BNP) levels before initiating ICIs [54,56]. Troponins and ECGs should be routinely monitored during treatment to detect early signs of cardiotoxicity. Additionally, a full cardiovascular assessment is recommended for long-term ICI therapy [54]. Baseline echocardiography should be performed with repeat imaging as needed for symptoms like chest pain or new heart failure signs. This comprehensive approach enables early detection and management of cardiovascular complications.

For patients receiving chemotherapeutic agents such as etoposide and cisplatin, along with radiotherapy, specific cardiac monitoring guidelines are limited. However, a high index of suspicion for CAD and acute coronary syndrome (ACS) should be maintained. Any symptoms indicative of ACS, including chest pain or dyspnea, require immediate evaluation and timely intervention to reduce the risk of severe cardiovascular events.

In conclusion, integrating cardiovascular monitoring into the standard care of patients undergoing lung cancer treatment is vital to mitigating therapy-related cardiac complications. Early recognition, preventive measures, and multidisciplinary collaboration can significantly improve survival rates and quality of life in this vulnerable patient population.

## Conclusions

CAD and lung cancer remain a significant public health concern and illustrate a bidirectional pathway. ACS in lung cancer are likely induced by treatment modalities. Although there have been cases of CAD in these therapies, there is a scarcity of data on accelerated CAD <1 year as in the case of our patient. It is important to have a high suspicion in these patients to promote early intervention and reduce mortality. Prevention and surveillance are key.
